# More Than Just a Complication: Post-ERCP Pancreatitis and Its Clinical Determinants in over 800 Procedures

**DOI:** 10.3390/jcm14196916

**Published:** 2025-09-29

**Authors:** Łukasz Nawacki, Agnieszka Bociek, Ada Bielejewska, Iwona Gorczyca-Głowacka, Stanisław Głuszek

**Affiliations:** 1 Collegium Medicum, Jan Kochanowski University, Aleja IX Wieków Kielc 19, 25-369 Kielce, Polandiwona.gorczyca@interia.pl (I.G.-G.); 2 Department of Anesthesiology and Intensive Care, Center of Postgraduate Medical Education, Orlowski Hospital, 00-416 Warsaw, Poland; 3 Holy Cross Cancer Hospital, Artwińskiego 3, 25-734 Kielce, Poland

**Keywords:** ERCP, PEP, post-ERCP pancreatitis, sphincterotomy, gallbladder stones, pancreatitis prevention

## Abstract

**Background:** Post-endoscopic retrograde cholangiopancreatography pancreatitis (PEP) remains the most frequent and clinically significant complication of ERCP, with a multifactorial etiology involving patient- and procedure-related risk factors. Despite preventive measures such as NSAIDs and peri-procedural stenting, the incidence of PEP has not substantially declined. We aimed to assess clinical determinants of PEP in a large real-world cohort treated with standardized procedural protocols. **Materials and Methods:** This retrospective single-center study analyzed 806 patients who underwent ERCP between January 2019 and December 2021. All procedures were performed by a single operator under general anesthesia with standardized prophylaxis (diclofenac 100 mg per rectum and cefazolin 2 g intravenously). Patients with delayed ERCP (>48 h from admission) or active acute pancreatitis were excluded. Logistic regression was used to identify independent predictors of PEP, hospital stay, and in-hospital mortality. **Results:** PEP occurred in 60 patients (7.4%). Independent risk factors included stenosis of the papilla of Vater (OR = 2.45; *p* = 0.025), gallbladder stones (OR = 2.66; *p* = 0.001), prior acute pancreatitis (OR = 2.72; *p* = 0.005), and sphincterotomy (OR = 2.53; *p* = 0.016). PEP was associated with longer hospitalization (MD = 4.5 days; *p* < 0.001) and increased in-hospital mortality (6.7% vs. 1.7%; *p* = 0.032). **Conclusion:** Stenosis of the papilla, gallbladder stones, prior acute pancreatitis, and sphincterotomy independently increased the risk of PEP, whereas older age, previous ERCP, and pancreaticoduodenal tumors were associated with a reduced risk. Despite standardized prophylaxis, PEP remains a relevant clinical concern. Identification of high-risk patients and individualized procedural planning are essential to minimizing complications.

## 1. Introduction

Acute pancreatitis (AP) is a common and potentially life-threatening gastrointestinal disorder characterized by local and systemic inflammation. It remains the leading cause of gastrointestinal-related hospital admissions in high-income countries, with an estimated annual incidence of 34 per 100,000 person-years [1]. In Poland, the incidence is approximately 72.1 per 100,000 population, with men more frequently affected, although women tend to present at an older age [2,3].

While most cases of AP are mild and self-limiting, approximately 20% progress to moderate or severe disease involving organ failure or pancreatic necrosis, with mortality rates reaching 20–40% [4]. Over the past decade, management of AP has shifted toward a multidisciplinary, minimally invasive approach focused on tailored care, which has led to improvements in both morbidity and mortality [5].

Endoscopic retrograde cholangiopancreatography (ERCP) is a key therapeutic and diagnostic procedure for biliary and pancreatic disorders. However, it is also the most common iatrogenic cause of acute pancreatitis. Post-ERCP pancreatitis (PEP) occurs in 1.3% to 15% of cases, and up to 25% in high-risk populations [6]. Though often mild, post-endoscopic retrograde cholangiopancreatography pancreatitis can progress to a severe form associated with significant morbidity and mortality, with reported death rates ranging from 3% to 5.6% depending on the population studied [7,8]. The pathogenesis of PEP is multifactorial, involving mechanical injury, hydrostatic pressure during contrast injection, premature enzymatic activation, and local inflammation [9,10]. Risk factors are typically classified into patient-related (e.g., female sex, younger age, sphincter of Oddi dysfunction) and procedure-related (e.g., difficult cannulation, precut sphincterotomy, pancreatic duct instrumentation, repeated attempts) [11,12,13]. Recent research also highlights the impact of anatomical and morphological features of the major duodenal papilla on cannulation difficulty and PEP risk [11].

Various preventive strategies have been investigated—including rectal NSAIDs, aggressive intravenous hydration, and prophylactic pancreatic stenting. However, results remain mixed. Several studies suggest that low-dose rectal diclofenac may be insufficient for effective prevention [14,15] and even high-dose indomethacin did not significantly reduce PEP incidence in a recent randomized trial [16]. Similarly, the utility of stents is debated, particularly in lower-risk populations [17].

While existing studies provide valuable insights, many are limited by sample heterogeneity or lack of adjustment for comorbid conditions and local clinical practice. Our retrospective analysis of over 800 ERCP procedures conducted at a single tertiary center offers an opportunity to reassess both established and emerging risk factors for PEP. Uniquely, our dataset includes granular patient information—including comorbidity profiles, procedural details, and post-procedural outcomes—allowing us to explore the influence of real-world factors such as indication for ERCP, type of intervention, and coexisting illnesses on both the incidence and severity of PEP. This may contribute to improved risk stratification and more individualized prophylactic strategies in clinical practice.

## 2. Materials and Methods

This retrospective study included patients hospitalized at a single tertiary care center between 1 January 2019, and 31 December 2021, who underwent endoscopic retrograde cholangiopancreatography (ERCP). The analysis was based on a local institutional database of ERCP procedures.

### 2.1. Inclusion and Exclusion Criteria

Patients were eligible for inclusion if ERCP was performed within 48 h of hospital admission. Individuals in whom the procedure was delayed beyond this window (>48 h) were excluded from the analysis to ensure clinical consistency and minimize confounding due to disease progression. The main reason for delays was institutional: ERCP in our center is available only during working hours (Monday to Friday, 08:00–13:00). In addition, some patients required postponement until correction of coagulation disorders or optimization of hemostasis. Less frequent reasons included stabilization of severe comorbid conditions (e.g., cardiac or respiratory instability) and resource-related factors such as operating room availability. Because these reasons may have systematically differentiated the excluded population, we acknowledge that this criterion could limit external validity. Additionally, patients who underwent ERCP due to active acute pancreatitis were excluded from the study, as the presence of pancreatitis at baseline could bias the evaluation of post-procedural outcomes, particularly the diagnosis of post-ERCP pancreatitis.

### 2.2. Procedure and Perioperative Management

All ERCPs were performed by the same experienced operator to eliminate inter-operator variability. Each procedure was carried out under general anesthesia. Standardized pre-procedural prophylaxis was applied across all cases:

- One hundred milligrams (100 mg) of diclofenac administered rectally;
- Two grams (2.0 g) of cefazolin administered intravenously, both given 30 min prior to the procedure.

### 2.3. Data Collection and Study Groups

Data were collected retrospectively from medical records and included the following:

- Demographics (age, sex);
- Clinical history and comorbidities (e.g., diabetes, obesity, liver disease, cancer, thyroid disorders);
- Indications for ERCP (e.g., gallbladder stones, bile duct stones, pancreaticobiliary tumors);
- Procedure details (sphincterotomy, stent placement);
- Symptoms and biochemical markers (e.g., pain, vomiting, CRP, amylase);
- Outcomes: post-ERCP pancreatitis (PEP), length of hospital stay, and in-hospital mortality.

Patients were stratified into subgroups based on the presence or absence of post-ERCP pancreatitis and further classified by severity of PEP, symptoms, and intervention type. Comorbidities such as diabetes, obesity, thyroid disorders, liver disease, kidney disease, and malignancy were recorded individually; however, we did not apply a validated composite index (e.g., Charlson Comorbidity Index or Elixhauser Comorbidity Index) to quantify overall comorbidity burden. Instead, comorbidity was analyzed descriptively at the level of individual conditions. We acknowledge this as a limitation, as the absence of a standardized index limits direct comparability with studies that report cumulative multimorbidity scores.

Unmeasured procedural variables: The source ERCP database did not prospectively capture several procedure-level covariates known to influence PEP risk, including cannulation difficulty and number of attempts, use of precut access, unintended pancreatic duct cannulation/injection or guidewire passages, procedure duration, and endoscopic morphology of the major duodenal papilla. Consequently, these variables were unavailable for analysis in the present study. To partially account for procedural complexity, we included sphincterotomy (yes/no) and indication for ERCP as proxies in all regression models. The associations reported should therefore be interpreted with awareness of potential residual confounding related to unmeasured procedural factors [7,11,12].

### 2.4. Endpoints

The primary endpoint was the incidence of post-ERCP pancreatitis (PEP). Secondary endpoints included the following:

- Identification of risk factors for PEP.
- Association of comorbidities with outcomes.
- Impact of intervention type (e.g., stenting, sphincterotomy).
- Length of hospitalization.
- In-hospital mortality.

### 2.5. Statistical Analysis

Quantitative variables were expressed as mean ± standard deviation (SD) or as median with interquartile range (IQR: 1st–3rd quartile), depending on data distribution. Normality was assessed using the Shapiro–Wilk test, together with skewness and kurtosis indicators, and homogeneity of variances was evaluated using Levene’s test.

Group comparisons were performed using parametric or non-parametric tests as appropriate: Student’s *t*-test or Welch’s *t*-test for normally distributed continuous variables, Mann–Whitney U test or Kruskal–Wallis test for non-normally distributed variables, and Pearson’s chi-square test or Fisher’s exact test for categorical variables. Effect sizes were reported as mean or median differences (continuous variables) or Cramer’s V (categorical variables), each with 95% confidence intervals (CIs).

To identify independent predictors of post-ERCP pancreatitis (PEP) and in-hospital mortality, we applied a two-step logistic regression approach. In the first step, variables with *p* < 0.25 in univariate analysis were considered as candidates. This relatively liberal cutoff was selected in line with the recommendations of Hosmer and Lemeshow (Applied Logistic Regression, 3rd ed.) [18], who suggest higher thresholds (up to 0.25) may help retain potentially important confounders that could become significant in a multivariable context. In the second step, a bidirectional stepwise selection procedure (both forward and backward) was performed, starting from the intercept-only model, with entry and removal thresholds set at *p* = 0.05 and *p* = 0.10, respectively.

Model calibration was assessed using the Hosmer–Lemeshow goodness-of-fit test, explanatory power was quantified with Nagelkerke’s R^2^, and multicollinearity was excluded by variance inflation factors (VIF; all < 1.5). Results are reported as odds ratios (ORs) with 95% CIs. All analyses were conducted using R software (version 4.4.2, R Foundation for Statistical Computing, Vienna, Austria; accessed 3 August 2025) [19].

### 2.6. Ethical Approval

This study was conducted in accordance with the principles of the Declaration of Helsinki and was approved by the Bioethical Committee of The Jan Kochanowski University in Kielce (approval no. 27/2023, dated 19 May 2023).

## 3. Results

### 3.1. General Characteristics of the Study Population

The study cohort consisted of 806 patients who underwent endoscopic retrograde cholangiopancreatography (ERCP) between 2019 and 2021. Among them, 436 (54.1%) were female, and the mean age of the entire group was 64.11 ± 15.15 years.

Regarding admission type, 307 patients (38.1%) were admitted electively, while 499 (61.9%) were admitted on an emergency basis.

The most common indication for ERCP was choledocholithiasis, reported in 371 patients (46.0%). Other frequent indications included the following:

- Pancreatic tumors: 92 patients (11.4%).
- Bile duct tumors: 75 patients (9.3%).
- Stenosis of the papilla of Vater: 58 patients (7.2%).
- Stent removal after previous ERCP: 45 patients (5.6%).
- Tumor of the papilla of Vater: 43 patients (5.3%).
- Bile duct stricture: 33 patients (4.1%).
- Gallbladder tumors: 24 patients (3.0%).
- Cholangitis: 23 patients (2.9%).
- Hilar liver tumors: 21 patients (2.6%).
- Postoperative complications: 16 patients (2.0%).
- Chronic pancreatitis: 3 patients (0.4%)
- Primary biliary cirrhosis and primary sclerosing cholangitis: 1 patient each (0.1%).

Additionally, 265 patients (32.9%) had gallbladder stones, 233 (28.9%) had post-cholecystectomy, and 208 (25.8%) had a history of previous ERCP. A prior diagnosis of acute pancreatitis was present in 92 patients (11.4%).

This section also captures the prevalence of comorbidities in the study group:

- Liver disease: 27 patients (3.3%).
- Non-pancreatic malignancies: 34 patients (4.2%).
- Diabetes: 144 patients (17.9%), with type 2 diabetes being the most prevalent (16.7%).
- Thyroid disorders: 36 patients (4.5%).
- Obesity: 50 patients (6.2%).

The baseline clinical characteristics, indications for ERCP, and comorbidities of the study population are summarized in Table 1. A more detailed comparison between the PEP and non-PEP groups, along with the identification of risk factors, will be presented in the subsequent sections.

### 3.2. Incidence and Clinical Characteristics of Post-ERCP Pancreatitis

Post-ERCP Pancreatitis (PEP) was diagnosed in 60 patients, representing 7.4% of the study population. Table 2 summarizes the clinical characteristics, symptomatology, treatment approaches, and severity grades among patients who developed PEP. This subgroup was clinically heterogeneous, with pain reported in nearly all cases (98.3%), followed by vomiting (51.7%) and muscular guarding (31.7%). All patients with PEP received both hydration and analgesic treatment, and 73.3% were additionally treated with antibiotics. According to the revised Atlanta classification, 46.7% of PEP cases were classified as mild, 31.7% as moderate, and 21.7% as severe. These data provide a foundation for further exploration of risk factors, outcomes, and predictors of severity within the cohort.

### 3.3. Comparison Between Patients with and Without Post-ERCP Pancreatitis

Among the 806 patients undergoing ERCP, significant differences were observed between those who developed PEP and those who did not (Table 3).

Patients who developed PEP were significantly younger, with a mean age difference of −4.76 years (95% CI: −9.47 to −0.05; *p* = 0.048). The type of ERCP indication was also significantly associated with PEP occurrence (*p* < 0.001). Specifically, choledocholithiasis (53.3% vs. 45.4%) and stenosis of the papilla of Vater (18.3% vs. 6.3%) were more frequent among patients with PEP. In contrast, other indications (including bile duct strictures, postoperative complications, cholangitis, hilar liver and gallbladder tumors, chronic pancreatitis, primary biliary cirrhosis, and primary sclerosing cholangitis) were less common among patients with PEP (28.3% vs. 48.3%). Despite statistical significance, the strength of association was weak (Cramér’s V = 0.14; 95% CI: 0.07–0.24).

Several clinical features also differed:

- Gallbladder stones were present in 58.3% of patients with PEP versus 30.8% in those without (*p* < 0.001; V = 0.15).
- A history of acute pancreatitis was more frequent among patients with PEP (21.7% vs. 10.6%; *p* = 0.017; V = 0.09).
- In contrast, pancreaticoduodenal tumors and previous ERCP were significantly less common among PEP cases (18.3% vs. 34.2% and 11.7% vs. 26.9%, respectively; *p* = 0.018 and *p* = 0.014).

Sphincterotomy was performed more frequently in the PEP group (85.0% vs. 64.6%; *p* = 0.002) and was associated with a weak effect (V = 0.11).

Patients with PEP experienced a significantly longer hospital stay, with a mean difference of 4.5 days (95% CI: 3.00–6.00; *p* < 0.001). Additionally, in-hospital mortality was higher in the PEP group (6.7% vs. 1.7%; *p* = 0.032), although the strength of association remained weak (V = 0.09).

### 3.4. Univariate Logistic Regression for PEP

Univariate analysis identified several clinical and procedural factors significantly associated with the risk of developing post-ERCP pancreatitis (PEP).

#### 3.4.1. Factors Associated with Increased Risk of PEP

- Stenosis of the papilla of Vater increased the odds of PEP more than twofold (OR = 2.48; 95% CI: 1.13–5.12; *p* = 0.018, reference: choledocholithiasis).
- Gallbladder stones were associated with over a threefold increase in odds (OR = 3.14; 95% CI: 1.85–5.42; *p* < 0.001).
- History of acute pancreatitis more than doubled the odds (OR = 2.34; 95% CI: 1.17–4.39; *p* = 0.011).
- Sphincterotomy was a strong predictor, increasing the odds of PEP by over three times (OR = 3.10; 95% CI: 1.58–6.84; *p* = 0.002).
- Longer hospital stay was associated with 9% higher odds of PEP per day (OR = 1.09; 95% CI: 1.06–1.13; *p* < 0.001).
- PEP itself was associated with higher in-hospital mortality (OR = 4.03; 95% CI: 1.11–11.82; *p* = 0.018).

#### 3.4.2. Factors Associated with Reduced Risk of PEP

- Older age was protective, with each additional year reducing the odds of PEP by 2% (OR = 0.98; 95% CI: 0.97–1.00; *p* = 0.020).
- Pancreaticoduodenal tumors were associated with a 57% reduction in odds (OR = 0.43; 95% CI: 0.21–0.82; *p* = 0.014).
- Previous ERCP procedures reduced the odds of PEP by 64% (OR = 0.36; 95% CI: 0.15–0.75; *p* = 0.012).
- Indications other than choledocholithiasis or papillary stenosis were also protective (OR = 0.50; 95% CI: 0.27–0.91; *p* = 0.025).

### 3.5. Multivariate Logistic Regression Analysis for PEP

Multivariate logistic regression analysis identified several independent risk factors significantly associated with the development of post-ERCP pancreatitis (PEP) (Table 4).

- Stenosis of the papilla of Vater as the indication for ERCP was associated with a twofold increase in PEP risk compared with choledocholithiasis (OR = 2.45; 95% CI: 1.08–5.26; *p* = 0.025).
- The presence of gallbladder stones increased the odds of PEP more than threefold (OR = 2.66; 95% CI: 1.50–4.79; *p* = 0.001).
- A history of acute pancreatitis was also a strong independent predictor, with a nearly threefold increase in risk (OR = 2.72; 95% CI: 1.31–5.34; *p* = 0.005).
- Sphincterotomy remained a significant procedural risk factor, associated with over a twofold increase in the odds of PEP (OR = 2.53; 95% CI: 1.25–5.71; *p* = 0.016).

The model’s performance, assessed using Nagelkerke’s R^2^, was 0.13, indicating moderate explanatory power and suggesting that additional unmeasured factors likely influence PEP risk. The Hosmer–Lemeshow goodness-of-fit test yielded a non-significant result (*p* = 0.259), indicating good model calibration. Evaluation of multicollinearity using variance inflation factors (VIFs) showed that all VIF values were below 1.5, confirming no multicollinearity among the included variables. Liver disease was retained in the final model by the stepwise procedure despite not reaching statistical significance (*p* = 0.069), as its inclusion improved overall model fit.

### 3.6. Post-ERCP Pancreatitis and Clinical Outcomes: Length of Hospital Stay and In-Hospital Mortality

Patients who developed post-ERCP pancreatitis (PEP) experienced significantly longer hospitalizations compared with those without PEP. The mean difference in length of stay was 4.50 days (95% CI: 3.00–6.00; *p* < 0.001), indicating a substantial increase in healthcare burden associated with this complication. This observation likely reflects the clinical consequences of PEP rather than factors that predispose to it.

In terms of in-hospital mortality, death was observed in 6.7% of patients with PEP compared with 1.7% in those without PEP. This difference was statistically significant (*p* = 0.032), although the strength of association was weak (Cramér’s V = 0.09; 95% CI: 0.00–0.20). However, the number of deaths was small (*n* = 17, including only 4 among patients with PEP), which limits the statistical power and precision of mortality-related estimates.

Furthermore, univariate logistic regression confirmed that the occurrence of PEP was significantly associated with increased odds of in-hospital death. The odds ratio for death among patients with PEP was 4.03 (95% CI: 1.11–11.82; *p* = 0.018), suggesting that PEP was an independent predictor of poor clinical outcome in this cohort.

These findings underscore the clinical importance of identifying high-risk patients and implementing effective preventive strategies for PEP, not only to reduce its incidence but also to mitigate its impact on patient survival and resource utilization.

### 3.7. Severity of PEP

Among the 60 patients who developed PEP (7.4% of the study population), severity according to the revised Atlanta classification was as follows: 28 (46.7%) mild, 19 (31.7%) moderate, and 13 (21.7%) severe. Clinical outcomes differed strikingly by severity grade. Patients with mild PEP had a median hospitalization of 8 days (IQR 6–9), similar to the non-PEP cohort, and none died during admission. Moderate PEP prolonged hospitalization substantially (median 15 days, IQR 12.5–17.5), reflecting greater resource use, though without in-hospital deaths. Severe PEP, while less common (1.6% of all ERCP procedures), was associated with the most unfavorable prognosis: median hospitalization reached 21 days (IQR 20–28), and all in-hospital deaths occurred in this subgroup (*n* = 4, 30.8%). Mortality in severe PEP therefore exceeded 15-fold the baseline risk observed in patients without PEP. These findings highlight the disproportionate clinical and prognostic burden carried by severe PEP, underscoring the importance of early recognition and targeted management in this subgroup.

## 4. Discussion

In this retrospective single-center study involving 806 patients undergoing ERCP, the incidence of post-ERCP pancreatitis (PEP) was 7.4%, aligning with previously reported rates in general populations. Multivariate logistic regression identified stenosis of the papilla of Vater, gallbladder stones, history of acute pancreatitis, and sphincterotomy as independent risk factors for PEP. In contrast, older age, pancreaticoduodenal tumors, previous ERCP, and other non-obstructive indications were associated with reduced risk. Patients who developed PEP had significantly longer hospital stays and a higher in-hospital mortality rate, underscoring the clinical relevance of early risk identification and preventive strategies. These findings contribute to a growing body of evidence supporting the need for individualized procedural planning and standardized PEP prevention protocols.

Our findings align with those of Nebiki et al. [17], who conducted a large multicenter prospective cohort study involving 807 patients undergoing endoscopic biliary stenting for malignant biliary obstruction and observed no protective effect of endoscopic sphincterotomy (ES) in preventing PEP. Similar to our cohort, the incidence of PEP in that population was 7.7%, and multivariate analysis showed no significant difference in PEP risk between patients with or without ES. In contrast, our single-center study demonstrated that sphincterotomy was independently associated with a more than twofold increased risk of PEP (OR = 2.53, 95% CI: 1.25–5.71). This discrepancy may be attributed to differences in patient selection. Nebiki et al. investigated a cohort restricted entirely to malignant biliary obstruction (100% of cases), whereas in our population, malignant indications (pancreatic tumors and bile duct tumors) accounted for only 20.7% (92 + 75 of 806 patients). The remaining majority of our cases were performed for choledocholithiasis, benign strictures, cholangitis, or other non-malignant conditions. This broader clinical spectrum may explain the different distribution of risk factors and outcomes. Notably, the work by El Nakeeb et al. [8] also supports the notion that certain procedural variables, including ES, increase the risk of post-procedural complications, although their analysis focused more broadly on risk factors for severe PEP. In their cohort, ES was more common among patients with severe forms of PEP, echoing our observation that the majority of PEP cases in our study occurred in patients who underwent sphincterotomy. These comparisons underscore the need to individualize the decision to perform ES based on patient-specific and procedural risk factors rather than employing it routinely as a preventive measure.

Our results are broadly consistent with those of Park et al. [20], who developed and validated two risk prediction models for PEP using a large Korean cohort of 1495 patients undergoing ERCP, reporting that younger age, female sex, history of acute pancreatitis, malignant biliary obstruction, and pancreatic sphincterotomy were independent risk factors for PEP. In our cohort, we similarly identified stenosis of the papilla of Vater, gallbladder stones, prior acute pancreatitis, and sphincterotomy as significant predictors, supporting the clinical relevance of these variables across diverse populations. Notably, while Park et al. emphasized the importance of post-procedural risk scoring (i.e., after procedural factors such as sphincterotomy are known), our findings suggest that several pre-procedural indicators—particularly gallbladder stones and history of pancreatitis—may help anticipate risk before the intervention and tailor prophylactic strategies accordingly. Additionally, in line with Park et al., our results also confirm that sphincterotomy remains a strong independent risk factor for PEP and should be weighed cautiously during therapeutic planning.

Further support comes from Cahyadi et al. [21], who reviewed the current strategies for PEP prevention and emphasized the multifactorial etiology of PEP involving patient- and procedure-related elements. They noted that prior pancreatitis, younger age, and biliary indications, especially difficult cannulation and instrumentation of the pancreatic duct, contribute significantly to PEP risk—elements which align well with the risk profile observed in our cohort. Importantly, our study adds to this by identifying that gallbladder pathology and stenosis of the papilla of Vater—frequently under-recognized in previous scoring models—are significantly associated with PEP, highlighting the need for localized validation and possibly model recalibration.

Another dimension worth considering in the pathogenesis of post-ERCP complications is the nature of post-procedural abdominal pain, particularly in patients not meeting diagnostic criteria for pancreatitis. Chen et al. [22] conducted a large-scale retrospective analysis of over 1200 ERCP procedures and identified independent risk factors for post-ERCP abdominal pain without PEP. These included younger age, primary ERCP, pancreatic guidewire passages, no papilla opening, and certain laboratory parameters (e.g., low WBC, low PLT, low γ-GT, and elevated albumin). Notably, some of these findings overlap with factors that were significantly associated with PEP in our analysis, especially younger age and pancreatic instrumentation. However, the authors emphasize that post-ERCP abdominal pain in the absence of PEP tends to occur earlier and is of lower frequency, supporting the need for nuanced clinical differentiation between benign post-interventional discomfort and early signs of PEP. This aligns with our finding that hospital stay was significantly longer in patients with confirmed PEP, indicating a more severe disease course.

Additionally, recent research by Wang et al. [11] focused on the papilla morphology, reporting that protruding and bulging papilla types were significantly associated with increased PEP incidence, likely due to anatomical challenges in cannulation and a higher risk of papillary trauma. While we did not include papilla morphology in our dataset, our findings that sphincterotomy and stenosis of the papilla of Vater were independently associated with increased PEP risk may reflect similar pathophysiological mechanisms—namely, mechanical difficulty during cannulation leading to pancreatic duct irritation or inadvertent trauma. Future prospective studies that incorporate endoscopic classification of the papilla (e.g., Type I–IV) could provide a more granular understanding of PEP risk stratification and may complement the clinical and procedural predictors identified in our model.

Our findings, particularly the identification of gallbladder stones, history of acute pancreatitis, and the role of sphincterotomy as significant risk factors for PEP, are consistent with emerging large-scale evidence on patient- and procedure-related predictors. The individual patient data meta-analysis by Sperna Weiland et al. [12] confirmed that even in patients receiving prophylaxis with rectal NSAIDs, factors such as difficult cannulation (RR 1.99), pancreatic contrast injection (RR 2.37), and history of PEP (RR 1.90) remained significant contributors to PEP risk despite standard preventive strategies. This underscores the multifactorial nature of PEP and supports the need for personalized prophylaxis, particularly in patients with complex anatomical or clinical profiles.

Stratification of PEP severity in our cohort adds prognostic context. Two-thirds of cases were mild, one-quarter were moderate, and fewer than 10% were severe, which mirrors distributions reported in previous studies [13,22]. Notably, all in-hospital deaths occurred among patients with severe PEP, highlighting the importance of differentiating severity grades when assessing outcomes. Predictive models should therefore consider not only the occurrence but also the severity of PEP.

In our cohort, patients who developed PEP were also significantly younger, more likely to have gallbladder stones and a history of pancreatitis, and more frequently underwent sphincterotomy, aligning with both studies. These convergent findings reinforce the notion that PEP risk stratification should not rely solely on procedural difficulty but also integrate patient comorbidities, previous disease history, and baseline laboratory status. The consistency across populations suggests that these risk factors may be robust and generalizable, although prospective validation—especially in real-world clinical settings—remains necessary.

In contrast to recent meta-analyses suggesting that diabetes may confer a protective effect against PEP, our findings did not demonstrate a significant association. One important explanation is that we were unable to account for glycemic control, which may substantially influence risk. Poorly controlled diabetes can promote microvascular damage, impaired tissue repair, and systemic inflammation, while long-standing, well-controlled diabetes may result in pancreatic atrophy and thereby reduce susceptibility to PEP. The absence of data on HbA1c, diabetes duration, and treatment regimens in our cohort, therefore, represents a key limitation. Jia et al. [10], in an analysis of more than 158,000 ERCP procedures, reported a lower incidence of PEP among patients with diabetes mellitus (OR 0.77; 95% CI: 0.63–0.94). In our study, diabetes was present in 17.9% of patients, and although evaluated in descriptive and univariate analyses, it was not significantly associated with PEP risk and was not retained in the multivariate model. This discrepancy may reflect population differences, variations in diabetes subtypes, or unmeasured confounders such as glycemic control, pancreatic atrophy, or disease duration. Future prospective studies integrating metabolic profiles and diabetes-specific variables are warranted to clarify the relationship between glucose metabolism and susceptibility to PEP.

Our findings align with the overarching challenge in optimizing strategies for PEP prevention, as highlighted in the comprehensive network meta-analysis by Arabpour et al. [23]. While our study focused on identifying clinical and procedural risk factors contributing to PEP in a real-world, heterogeneous population, their meta-analysis dissected the efficacy of various prophylactic strategies, notably emphasizing the superiority of combined regimens—particularly indomethacin with normal saline hydration (I + NS) or pancreatic duct stenting (I + S)—in reducing the incidence of PEP in both average- and high-risk groups. Interestingly, while our data underscored the role of specific clinical features, such as gallbladder stones, history of acute pancreatitis, or the use of sphincterotomy in modulating PEP risk, Arabpour et al. pointed to variability in patient risk profiles and procedural techniques as critical determinants in prophylaxis efficacy. This reinforces the notion that a personalized approach to both risk assessment and prophylaxis selection is essential. Moreover, their analysis showed that the prevention of moderate-to-severe PEP remains particularly challenging, with only a few interventions (notably I + LR and stents) demonstrating significant efficacy, which further substantiates the need for stratified prophylaxis depending on predicted severity.

Our findings are consistent with the broader conclusions of the systematic review by Pekgöz et al. [6], who emphasized that PEP remains the most common and clinically significant complication of ERCP, with multifactorial etiology encompassing patient-related, procedure-related, and operator-dependent risk factors. The authors identified female sex, young age, history of pancreatitis, normal serum bilirubin, and difficult cannulation as prominent contributors to PEP risk. Although our study did not assess sex or cannulation difficulty directly, we confirmed that younger age and prior acute pancreatitis were independently associated with increased PEP risk. Moreover, Pekgöz et al. reinforced the role of sphincterotomy as a key procedural risk factor, particularly in patients with anatomically challenging papillae or pancreatic outflow obstruction—a finding that echoes our observation that sphincterotomy was independently associated with more than a twofold increase in PEP risk. Importantly, their review highlighted the limited efficacy of single-agent pharmacologic prophylaxis in moderate- to high-risk patients, aligning with our conclusion that pre-procedural identification of risk factors must be followed by targeted, multifaceted prevention strategies to effectively reduce the burden of PEP.

Differences between our findings and those of previous studies likely reflect variations in patient populations, procedural practices, and definitions of comorbidities. For example, while some large meta-analyses have suggested that diabetes may be associated with a reduced risk of PEP [10], our analysis did not confirm this relationship. Potential explanations include differences in the prevalence of type 1 versus type 2 diabetes, variations in glycemic control, and the degree of pancreatic atrophy associated with long-standing diabetes, none of which were captured in our dataset. Furthermore, international cohorts often include heterogeneous operator experience and a wider spectrum of prophylactic regimens, whereas in our study, all procedures were conducted by a single experienced endoscopist using standardized prophylaxis. These contextual differences may account for the observed discrepancies and highlight the importance of validating PEP risk factors in diverse healthcare settings.

### Strengths and Limitations

Our study has several important strengths but also notable limitations that should be considered.

First, our dataset did not include several procedure-level determinants of PEP, such as cannulation difficulty, number of attempts, precut use, unintended pancreatic duct instrumentation, and papillary morphology. These factors are consistently linked to PEP risk in prospective cohorts and meta-analyses [7,11,12]. Due to the retrospective design, they were not described systematically in operative protocols and could not be analyzed. Although we adjusted for available procedural proxies (sphincterotomy and indication), residual confounding remains possible. For example, the observed association between sphincterotomy and PEP may partly reflect underlying cannulation difficulty. Future prospective registries should incorporate standardized metrics (e.g., cannulation time, number of guidewire passages, papilla morphology classification) to refine risk stratification and improve model calibration.

Second, while we analyzed individual comorbidities, we did not apply a validated multimorbidity index such as the Charlson Comorbidity Index (CCI) or Elixhauser Comorbidity Measure. The absence of a composite score limited our ability to assess the cumulative effect of comorbidity burden on PEP risk and outcomes. Future studies should integrate such validated indices, as they provide more robust adjustment and facilitate cross-cohort comparisons.

Third, our prophylactic regimen was uniform and limited to diclofenac, with all patients receiving the same non-steroidal anti-inflammatory drug. This homogeneity reduces variability but precludes evaluation of alternative prophylactic strategies such as indomethacin, aggressive periprocedural hydration, or pancreatic duct stenting, which are also recommended in guidelines [21]. Thus, our findings should be interpreted in the context of standardized diclofenac prophylaxis, and future studies should compare these approaches to identify the most effective prevention strategy in real-world practice.

Fourth, the explanatory power of our regression model was modest (Nagelkerke R^2^ = 0.13). While calibration was acceptable, the low R^2^ suggests that many relevant determinants of PEP were not captured in our dataset. Missing variables such as procedural details, comorbidity indices, and biochemical markers may account for the unexplained variance. This highlights the need for more granular data in prospective multicenter registries.

Fifth, our conclusions regarding in-hospital mortality should be interpreted cautiously. Only seventeen patients died overall, and just four of these deaths occurred among patients with PEP. Such a small event number reduces statistical power, limits the precision of mortality estimates, and increases the risk of unstable effect sizes. Larger multicenter cohorts are necessary to validate mortality outcomes associated with PEP.

Sixth, our study was restricted to in-hospital outcomes. We did not collect long-term follow-up data such as recurrent pancreatitis, late complications, or need for repeat ERCP. This limitation stems not only from the retrospective design but also from our institutional context: as a tertiary referral center, many patients are re-hospitalized in smaller regional hospitals when complications occur, making systematic follow-up infeasible; consequently, our dataset reflects only the immediate clinical course. Long-term outcomes remain clinically important, and prospective multicenter studies with coordinated follow-up are needed to provide a more comprehensive assessment of PEP impact.

Seventh, while the single-center, single-operator design improves internal validity by ensuring procedural consistency, it limits external validity. Referral patterns, operator practices, and prophylactic strategies differ across institutions. Our findings may therefore not be generalizable to other populations. Additionally, although diabetes was not associated with PEP in our cohort, contrasting with the meta-analysis by Jia et al. [10], we were unable to adjust for glycemic control, disease duration, or pancreatic morphology, which could modify this relationship. Future research should integrate metabolic parameters to clarify the association between glucose metabolism and PEP risk.

We note that liver disease (*p* = 0.069) was retained in the multivariate model. This reflects our modeling approach: variables with univariate *p* < 0.25 were eligible for entry, and stepwise selection retained liver disease based on its contribution to model fit. Although not statistically significant at α = 0.05, its inclusion aligns with Hosmer and Lemeshow’s recommendations to avoid prematurely excluding potentially relevant predictors.

While the limitations outlined above should be acknowledged, our study also possesses several notable strengths that enhance the reliability and clinical relevance of the findings. These strengths relate not only to the size and composition of the cohort but also to the uniformity of procedures, the breadth of clinical variables analyzed, and the methodological rigor applied in the statistical approach. Together, they provide a solid foundation for interpreting the results and for informing future research directions.

First, the relatively large and homogeneous patient cohort substantially increases the reliability of our findings. More than 800 ERCP procedures were analyzed, making this one of the largest single-center series of its kind. Importantly, all procedures were performed by a single highly experienced endoscopist in a tertiary referral center, under standardized anesthesia and with uniform prophylaxis (rectal diclofenac and cefazolin). This unique setting markedly reduces variability associated with operator skill, endoscopic technique, anesthesia, and prophylactic regimen—sources of heterogeneity that often complicate interpretation in multicenter cohorts.

Second, our study provides a rich clinical characterization of both patient-related and procedure-related variables. We included underreported factors such as stenosis of the papilla of Vater and a history of acute pancreatitis, which are inconsistently captured in other studies but proved to be important risk determinants in our analysis. We also assessed protective factors (older age, previous ERCP, pancreaticoduodenal tumors), offering a more nuanced risk profile than many prior reports that focus predominantly on risk factors alone. This dual perspective is clinically valuable, as it can inform both patient selection and preventive strategies.

Third, compared with previously published risk stratification models [20,21], our study incorporated a broader range of real-world variables. These included not only established predictors such as gallstones, prior acute pancreatitis, and sphincterotomy, but also comorbid conditions, prior interventions, and detailed ERCP indications. This comprehensive approach allowed us to identify independent risk factors while also highlighting conditions associated with lower risk, thereby broadening the clinical applicability of our findings.

Fourth, our statistical analysis adhered to methodological best practices. We performed structured, stepwise logistic regression with prespecified entry/removal thresholds, evaluated collinearity with variance inflation factors, and assessed model calibration using the Hosmer–Lemeshow test. We reported effect sizes, odds ratios, and model performance measures (Nagelkerke R^2^), enabling both statistical interpretation and clinical translation. This transparent and rigorous approach is consistent with standards recommended in recent ERCP research and meta-analyses [12,23].

Fifth, by focusing on a single tertiary center, our study benefits from data consistency and internal validity. Patient selection, peri-procedural management, anesthesia protocols, and operator technique were highly standardized, minimizing noise from inter-institutional and inter-operator variability. This design strengthens causal inference within the context of our cohort and ensures that the associations observed are less likely to be driven by procedural heterogeneity.

Finally, our study demonstrated the clinical impact of PEP in real-world practice. We confirmed that PEP significantly prolongs hospitalization and increases mortality, and we provided a granular analysis of severity, showing that deaths were confined to the severe PEP subgroup. These insights underscore the importance of both risk stratification and early detection.

Taken together, these strengths highlight the value of our study: it combines a large, well-characterized cohort with rigorous methodology, contributes novel insights into underreported risk and protective factors, and provides clinically relevant outcomes. Our findings can therefore serve as a robust reference point for future multicenter prospective studies aiming to refine PEP risk prediction and prevention strategies.

## 5. Conclusions

In this single-center retrospective study, the incidence of post-ERCP pancreatitis (PEP) was 7.4%, aligning with international benchmarks. We identified stenosis of the papilla of Vater, gallbladder stones, prior acute pancreatitis, and sphincterotomy as independent risk factors for PEP, while older age, previous ERCP, and pancreaticoduodenal tumors were associated with a reduced risk. Importantly, PEP was linked to significantly longer hospitalization and higher in-hospital mortality, underscoring its clinical and economic impact.

Our findings reinforce the need for early risk stratification, incorporating both patient- and procedure-related variables. Even in settings with standardized prophylaxis, individualized procedural planning remains critical for reducing complications. Future prospective studies are warranted to validate these predictors and to optimize PEP prevention strategies through integrated clinical, procedural, and metabolic profiling.

## Figures and Tables

**Table 1:** Baseline characteristics, ERCP indications, and comorbidities of the study population (*n* = 806).

Characteristic	Value
Sex (female), *n* (%)	436 (54.1)
Age, years, M ± SD	64.11 ± 15.15
Admission type—elective, *n* (%)	307 (38.1)
Admission type—emergency, *n* (%)	499 (61.9)
Indications, *n* (%)	
Choledocholithiasis	371 (46.0)
Pancreatic tumor	92 (11.4)
Bile duct tumor	75 (9.3)
Stenosis of the papilla of Vater	58 (7.2)
Status post ERCP—stent removal	45 (5.6)
Tumor of the papilla of Vater	43 (5.3)
Bile duct stricture	33 (4.1)
Gallbladder tumor	24 (3.0)
Cholangitis	23 (2.9)
Hilar liver tumor	21 (2.6)
Postoperative complications	16 (2.0)
Chronic pancreatitis	3 (0.4)
Primary biliary cirrhosis	1 (0.1)
Primary sclerosing cholangitis	1 (0.1)
Gallbladder stones, *n* (%)	265 (32.9)
Choledocholithiasis (bile duct stones), *n* (%)	371 (46.0)
Post cholecystectomy status, *n* (%)	233 (28.9)
Pancreaticoduodenal tumor, *n* (%)	266 (33.0)
Previous ERCP, *n* (%)	208 (25.8)
History of acute pancreatitis, *n* (%)	92 (11.4)
Liver disease, *n* (%)	27 (3.3)
Malignancy other than pancreaticoduodenal tumor, *n* (%)	34 (4.2)
Diabetes, *n* (%)	144 (17.9)
Diabetes by type, *n* (%)	
Type 1	6 (0.7)
Type 2	135 (16.7)
Type 3	3 (0.4)
None	662 (82.1)
Thyroid disease, *n* (%)	36 (4.5)
Thyroid disease by type, *n* (%)	
Hyperthyroidism	14 (1.7)
Hypothyroidism	13 (1.6)
Nontoxic goiter	9 (1.1)
None	770 (95.5)
Obesity, *n* (%)	50 (6.2)
Sphincterotomy, *n* (%)	533 (66.1)
Endoscopic stent, *n* (%)	526 (65.3)
Endoscopic stent by type, *n* (%)	
Plastic stent	399 (49.5)
Self-expandable stent	57 (7.1)
Stent removal	70 (8.7)
None	280 (34.7)
Post-endoscopic pancreatitis, *n* (%)	60 (7.4)
Asymptomatic hyperamylasemia, *n* (%)	26 (3.2)
Length of hospital stay, days, Me (Q1; Q3)	7.00 (4.00; 11.00)
Death, *n* (%)	17 (2.1)

**Table 2:** Incidence, clinical presentation, treatment, and severity of post-ERCP pancreatitis (*n* = 60).

Characteristic	Value
Symptoms, *n* (%)	
Pain	59 (98.3)
Vomiting	31 (51.7)
Muscular guarding	19 (31.7)
Amylase, U/L, Me (Q1; Q3) (normal value: 30–100 U/l)	1244.50 (704.50;1683.50)
CRP, mg/L, Me (Q1; Q3) (normal value: 0–5 mg/L)	55.00 (29.75;101.00)
Therapy, *n* (%)	
Hydration	60 (100.0)
Analgesics	60 (100.0)
Antibiotics	44 (73.3)
Severity Grade, *n* (%)	
Mild	28 (46.7)
Moderate	19 (31.7)
Severe	13 (21.7)

**Table 3:** Comparison of demographic, clinical, and procedural characteristics between patients with and without post-ERCP pancreatitis (PEP).

Characteristic	Post-Endoscopic Pancreatitis				Univariate Logistic Regression for Post-Endoscopic Pancreatitis		
	Yes (*n* = 60)	No (*n* = 746)	MD/V (95% CI)	*p*	OR	95% CI for OR	*p*
Sex (female), *n* (%)	33 (55.0)	403 (54.0)	0.01 (0.00; 0.08)	0.991	1.04	0.61–1.78	0.884
Age, years, M ± SD	59.70 ± 17.78	64.46 ± 14.87	−4.76 (−9.47; −0.05)	**0.048**	0.98	0.97–1.00	**0.020**
Admission Elective, *n* (%)	19 (31.7)	287 (38.5)	0.04 (0.00; 0.10)	0.365	0.74	0.41–1.28	0.298
Admission Emergency, *n* (%)	41 (68.3)	458 (61.4)	0.04 (0.00; 0.11)	0.354	1.36	0.78–2.43	0.288
Indications, *n* (%)							
Choledocholithiasis	32 (53.3)	339 (45.4)	0.14 (0.07; 0.24)	**<0.001**	*ref.*		
Stenosis of the papilla of Vater	11 (18.3)	47 (6.3)			2.48	1.13–5.12	**0.018**
Other *	17 (28.3)	360 (48.3)			0.50	0.27–0.91	**0.025**
Gallbladder stones, *n* (%)	35 (58.3)	230 (30.8)	0.15 (0.08; 0.22)	**<0.001**	3.14	1.85–5.42	**<0.001**
Choledocholithiasis (bile duct stones), *n* (%)	32 (53.3)	339 (45.4)	0.04 (0.00; 0.11)	0.296	1.37	0.81–2.34	0.240
Post cholecystectomy status, *n* (%)	13 (21.7)	220 (29.5)	0.05 (0.00; 0.11)	0.255	0.66	0.34–1.21	0.201
Pancreaticoduodenal tumor, *n* (%)	11 (18.3)	255 (34.2)	0.09 (0.03; 0.15)	**0.018**	0.43	0.21–0.82	**0.014**
Previous ERCP, *n* (%)	7 (11.7)	201 (26.9)	0.09 (0.04; 0.14)	**0.014**	0.36	0.15–0.75	**0.012**
History of acute pancreatitis, *n* (%)	13 (21.7)	79 (10.6)	0.09 (0.01; 0.18)	**0.017**	2.34	1.17–4.39	**0.011**
Liver disease, *n* (%)	4 (6.7)	23 (3.1)	0.05 (0.00; 0.15)	0.134	2.25	0.64–6.09	0.148
Malignancy other than pancreaticoduodenal tumor, *n* (%)	3 (5.0)	31 (4.2)	0.01 (0.00; 0.09)	0.735	1.21	0.29–3.54	0.755
Diabetes, *n* (%)	14 (23.3)	130 (17.4)	0.04 (0.00; 0.12)	0.330	1.44	0.74–2.64	0.253
Diabetes by type, *n* (%)							
Type 1	0 (0.0)	6 (0.8)	0.08	0.222	0.00	-	0.983
Type 2	13 (21.7)	122 (16.4)			1.43	0.72–2.65	0.280
Type 3	1 (1.7)	2 (0.3)			6.70	0.31–71.16	0.123
None	46 (76.7)	616 (82.6)			*ref.*		
Thyroid disease, *n* (%)	3 (5.0)	33 (4.4)	0.01 (0.00; 0.08)	0.745	1.14	0.27–3.30	0.835
Thyroid disease by type, *n* (%)							
Hyperthyroidism	1 (1.7)	13 (1.7)	0.05 (0.03; 0.15)	0.465	0.96	0.05–4.96	0.971
Hypothyroidism	2 (3.3)	11 (1.5)			2.27	0.35–8.73	0.293
Nontoxic goiter	0 (0.0)	9 (1.2)			0.00	-	0.986
None	57 (95.0)	713 (95.6)			*ref.*		
Obesity, *n* (%)	3 (5.0)	47 (6.3)	0.01 (0.00; 0.07)	>0.999	0.78	0.19–2.23	0.689
Sphincterotomy, *n* (%)	51 (85.0)	482 (64.6)	0.11 (0.05; 0.17)	**0.002**	3.10	1.58–6.84	**0.002**
Endoscopic stent, *n* (%)	36 (60.0)	490 (65.7)	0.03 (0.00; 0.10)	0.454	0.78	0.46–1.36	0.375
Endoscopic stent by type, *n* (%)							
Plastic stent	32 (53.3)	367 (49.2)	0.07 (0.04; 0.13)	0.248	0.93	0.54–1.63	0.797
Self-expandable stent	2 (3.3)	55 (7.4)			0.39	0.06–1.36	0.207
Stent removal	2 (3.3)	68 (9.1)			0.31	0.05–1.09	0.121
None	24 (40.0)	256 (34.3)			*ref.*		
Asymptomatic hyperamylasemia, *n* (%)	0 (0.0)	26 (3.5)	0.05 (0.04; 0.06)	0.250	0.00	-	0.984
Length of hospital stay, days, Me (Q1; Q3)	11.50 (8.00; 19.00)	7.00 (4.00; 11.00)	4.50 (3.00; 6.00)	**<0.001**	1.09	1.06–1.13	**<0.001**
Death, *n* (%)	4 (6.7)	13 (1.7)	0.09 (0.00; 0.20)	**0.032**	4.03	1.11–11.82	**0.018**

M—mean, SD—standard deviation, Me—median, Q1—1st quartile, Q3—3rd quartile, MD—mean/median difference (yes vs. no), presented for age and length of stay, V—Cramer V coefficient, presented for all categorical variables, CI—confidence interval, OR—odds ratio as outcome of univariate logistic regression analysis. Comparison between patients with post-endoscopic pancreatitis and patients without post-endoscopic pancreatitis was performed using the t Welch test (age), Mann–Whitney U test (length of hospital stay), Fisher’s exact test (malignancy other than pancreaticoduodenal tumor, type of diabetes, thyroid disease, type of thyroid disease, obesity, asymptomatic hyperamylasemia, and death) and Pearson’s chi-square test (all other variables), as appropriate. * Other indications include bile duct strictures, postoperative complications, cholangitis, hilar liver tumors, gallbladder tumors, chronic pancreatitis, primary biliary cirrhosis, and primary sclerosing cholangitis.

**Table 4:** Multivariate logistic regression model for predictors of post-ERCP pancreatitis (PEP).

Characteristic	OR	95% for OR	*p*
Indications			
Choledocholithiasis	*ref.*		
Stenosis of the papilla of Vater	2.45	1.08–5.26	**0.025**
Other *	0.69	0.36–1.30	0.260
Gallbladder stones	2.66	1.50–4.79	**0.001**
History of acute pancreatitis	2.72	1.31–5.34	**0.005**
Liver disease	2.88	0.80–8.30	0.069
Sphincterotomy	2.53	1.25–5.71	**0.016**

* Other indications include bile duct strictures, postoperative complications, cholangitis, hilar liver tumors, gallbladder tumors, chronic pancreatitis, primary biliary cirrhosis, and primary sclerosing cholangitis.

## Data Availability

Data are available from the corresponding author upon reasonable request.
